# Transmission events revealed in tuberculosis contact investigations in London

**DOI:** 10.1038/s41598-018-25149-6

**Published:** 2018-04-27

**Authors:** Sean M. Cavany, Emilia Vynnycky, Tom Sumner, Neil Macdonald, H. Lucy Thomas, Jacqui White, Richard G. White, Helen Maguire, Charlotte Anderson

**Affiliations:** 1 TB Modelling Group, TB Centre and CMMID, Faculty of Epidemiology and Population Health, London School of Hygiene and Tropical Medicine, London, UK; 2 Statistics, Modelling and Economics Department, Public Health England, London, UK; 3 Respiratory Diseases Department, Public Health England, London, UK; 4 Field Epidemiology Service, Public Health England, London, UK; 5 North Central London TB Service, Whittington Health NHS Foundation Trust, London, UK; 6 Institute for Global Health, University College London, London, UK

## Abstract

Contact tracing is a key part of tuberculosis prevention and care, aiming to hasten diagnosis and prevent transmission. The proportion of case-contact pairs for which recent transmission occurred and the typical timespans between the index case and their contact accessing care are not known; we aimed to calculate these. We analysed individual-level TB contact tracing data, collected in London from 20/01/2011-31/12/2015, linked to tuberculosis surveillance and MIRU-VNTR 24-locus strain-typing information. Of pairs of index cases and contacts diagnosed with active tuberculosis, 85/314 (27%) had strain typing data available for both. Of these pairs, 79% (67/85) shared indistinguishable isolates, implying probable recent transmission. Of pairs in which both contact and the index case had a social risk factor, 11/11 (100%) shared indistinguishable isolates, compared to 55/75 (75%) of pairs in which neither had a social risk factor (*P* = 0.06). The median time interval between the index case and their contact accessing care was 42 days (IQR: 16, 96). As over 20% of pairs did probably not involve recent transmission between index case and contact, the effectiveness of contact tracing is not necessarily limited to those circumstances where the index case has transmitted disease to their close contacts.

## Introduction

Contact tracing, the systematic screening of contacts of tuberculosis (TB) cases, is an important part of TB control in the UK and other high-income, low-incidence countries, and is highlighted as a key element of the Public Health England (PHE)/National Health Service (NHS) England collaborative tuberculosis strategy^1^. It aims to reduce transmission from and morbidity in contacts with active TB, and find contacts with latent *M. tuberculosis* infection that are eligible for preventive therapy^2^. London accounts for nearly 40% of England’s TB cases^3^, and during 20/01/2011-31/12/2015, 13 692 TB cases were notified in London, with a median of four contacts identified per case^3,4^.

If a contact is diagnosed with active TB, there may have been recent transmission between index case and contact, or the contact may have been infected, recently or historically, by another source. Understanding the extent to which cases found through contact tracing are due to recent transmission shows the value of contact tracing in interrupting ongoing transmission. Where the proportion of cases identified due to recent transmission is high, this is more likely to reflect active and ongoing transmission. Reducing the time between the index case and their contact accessing care means earlier diagnosis of an active case, benefitting the individual and reducing the risk of onward transmission from an infectious source. Whilst around 5% of cases in London are found through contact tracing [unpublished data], less is known if those cases are due to recent transmission, nor of the typical timescales (such as the median time between the index case and their contact accessing care) involved in contact investigations.

Since 2010 in the UK, isolates from culture confirmed TB cases have been routinely strain-typed using 24-loci mycobacterial-interspersed-repetitive-units – variable-number-tandem-repeats (MIRU-VNTR) strain-typing^5^. Using strain-typing data, transmission between cases is considered not to have occurred if their isolates are distinct and probable if the isolates are indistinguishable and supported by evidence of contact between cases. Previous studies using strain-typing data in the United States found that around 70% of contacts with TB may have been infected by or have infected their index case^6–8^, but no studies have estimated this in the UK. Whilst a recent study in France estimated the time between index case notification and the contact being screened to be 48 days^9^, to our knowledge no studies have directly estimated the time interval between index case notification and diagnosis of TB in a contact in the UK.

We aimed to describe the extent of transmission that was identified through contact tracing, and the time taken from index case identification to finding the active case among their contacts. This was in order to provide TB services with evidence for the value of contact investigations, and where efforts might be targeted or strengthened in order to give the biggest benefit.

Our first objective was to estimate the proportion of index case-contact pairs for whom probable recent transmission had occurred, and determine factors associated with differences in this proportion. The second objective was to estimate the time interval between the index case and the contact accessing care, as a proxy measure for contact investigation length, and determine which factors are associated with longer or shorter intervals. An additional aim of the study was to understand whether the patient characteristics of those contact tracing pairs found to be due to recent transmission were also common among the pairs tracing for whom investigations are longer.

## Methods

### Dataset and inclusion criteria

The primary data source was the London TB Register (LTBR; a web-based register containing demographic and clinical data on all TB cases notified in London since 2002). TB cases are notified in England either if they are culture confirmed, or based on the clinician’s decision to treat with a full course of anti-TB therapy. From 2012, the LTBR has incorporated data on contact tracing from ‘cohort review’; this is a quarterly case management and contact tracing appraisal conducted by clinical staff for TB cases, introduced incrementally across London from 2010^10^. This paper utilizes contact tracing data collected as part of cohort review, linked to surveillance data from the LTBR and strain typing data held by PHE.

Contacts identified during contact investigations and diagnosed with active TB are linked in the LTBR to the index case of the investigation. For the period of the study (20/01/2011-31/12/2015) contact tracing was conducted according to national guidance CG117^11^, which recommended screening household and other close contacts of all cases. During the study period, contact investigations began immediately after the diagnosis of the index case, whereupon the nurse asked the case for a list of close contacts. Screening begins with symptom-screen; for asymptomatic contacts this is followed by a tuberculin skin test (TST) or interferon-gamma release assay (IGRA) in those aged under 35 years and consideration of a chest X-ray (CXR) in those aged 35 years and over. Those with a positive symptom-screening, TST/IGRA result or CXR are evaluated for signs of active TB. Those with LTBI are considered for preventive therapy.

The study population included any pair for which the index case was notified in the study period and had at least one contact diagnosed with active TB. Pairs were excluded if the linked contact began their current episode of care prior to their index case. For the first objective, analysis was further limited to pairs for which both contact and index case had strain typing results.

In London, isolates of culture-confirmed TB cases are typed at the PHE Mycobacterium Reference Laboratory, with results matched with surveillance data using the Enhanced Matching System (EMS)^12^. Isolates are defined as indistinguishable if at least one in the cluster had 24 loci typed and all others had 23 or 24 loci typed and matched in all typed loci.

Within the LTBR, the episode of care start date is the date when a patient was first seen by the clinic at which they were notified, and is a mandatory field recorded for all patients.

### Analysis

We took the following approach for the two objectives:

1. To determine the proportion of strain typed pairs for whom index and contact had indistinguishable isolates. This was stratified by country of birth, site of disease & smear status, social risk factors (current or history of: imprisonment, drug or alcohol misuse and/or homelessness), age and sex, based on attributes of the index, contact or shared by both. We evaluated the sensitivity of these results to excluding contacts not recorded as presenting through contact screening, and to including pairs for whom the contact accessed care first. There were four instances when the same contact with TB was named by two different index cases; we evaluated the sensitivity of results to the exclusion of these links so that each contact only appeared in one pair.
2. To estimate the median and distribution of the time interval between episode of care start dates of index and contact(s). We measured the interval in days between episode of care start date of the index case and contact, and explored whether the factors mentioned in objective one were associated with longer time intervals, in each instance adjusted for the site of disease of the index. Differences in medians were assessed for significance using Mood’s median test.

### Software

All data were analysed using Microsoft Excel 14.0 and Stata 13.1.

### Data availability

Aggregate data that support the findings of this study are available on reasonable request from the corresponding author (SMC). The individual level data generated and analysed during the current study are not publicly available as the data were collected in adherence with the legal framework governing use of confidential personally identifiable information.

### Ethics

Ethical approval was not required. The data analysed were routinely collected surveillance data held by PHE under Section 251 of the NHS Act 2006. All records were anonymised before analysis.

## Results

### Comparison of excluded and included data

**Figure 1:**
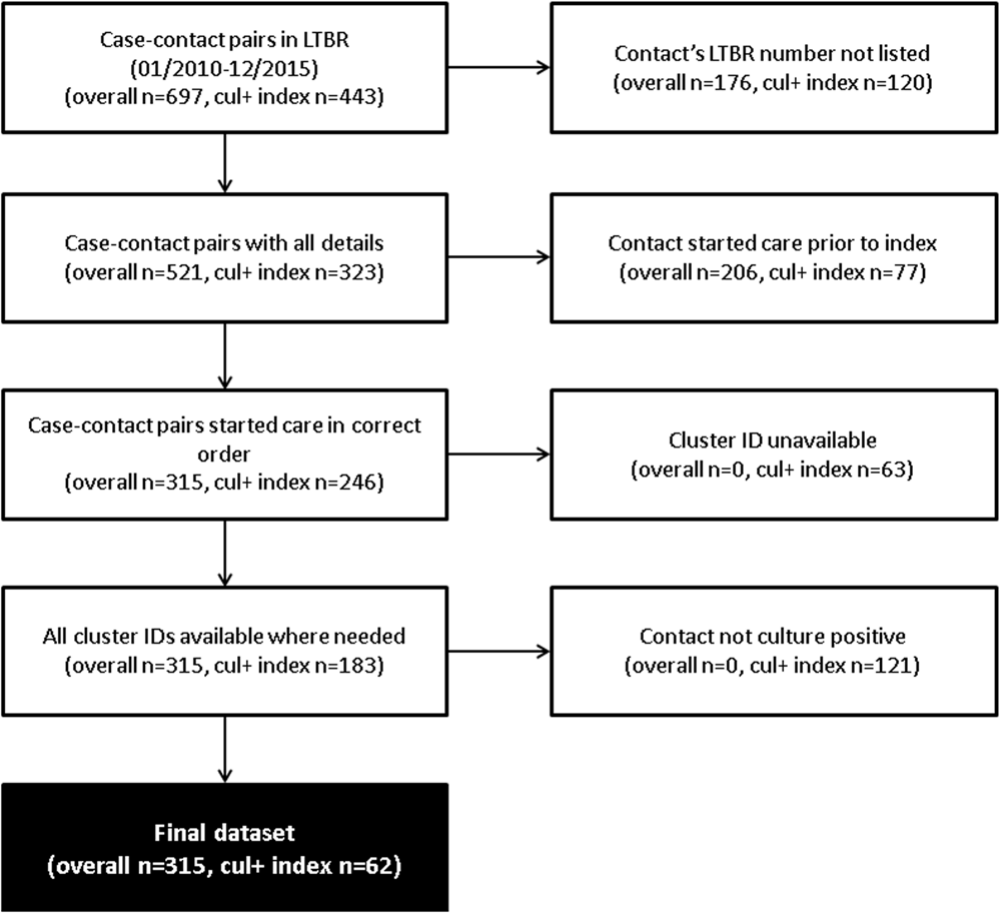
Flowchart of included and excluded case-contact pairs, for objectives one (‘typed index’) and objective two (‘overall’). Note that boxes three and four on the right were not exclusion criteria for objective two.

There were 451 cases of TB in the study period recorded as having one or more contacts diagnosed with TB (286 when restricting to strain typed pairs), resulting in 697 potential case-contact pairs (406 when restricting to strain typed pairs) (Fig. 1). After applying the inclusion criteria, 85 pairs (21%) were included in the analyses for objective one and 314 pairs (45%) for objective two, corresponding to 81/286 (28%) and 247/451 (55%) of all strain-typed index cases and all index cases, respectively. There were 44 index cases included in more than one case- contact pair overall, and three in more than one strain-typed pair.

In the analyses for each objective, a varying number of pairs were also removed where there were insufficient data on the demographic trait or clinical characteristic of interest. Those included in analysis for objective one had a different ethnic profile to those excluded (Table 1). Index cases included for objective two were more likely to be male, have pulmonary disease or be 15 years old or above, than those excluded (Table 1). As children are less likely to be culture positive than adults, adults were over-represented in both included and excluded pairs for analysis of objective one relative to objective two.

**Table 1:** Comparison of characteristics of index cases included in the analyses and those with one or more contacts who were diagnosed with TB but were excluded from the analyses.

Factor		Analyses of indistinguishable isolates of case-contact-pairs (objective 1)			Analyses of time between episode of care start date of index case and contact (objective 2)		
		Number included (%)	Number excluded (%)	p-value	Number included (%)	Number excluded (%)	p-value
Total		81 (28%)	205 (72%)	N/a	247 (55%)	204 (45%)	N/a
UK-born?	Yes	28 (35%)	50 (24%)	0.22	82 (33%)	70 (34%)	0.68
	No	44 (54%)	127 (62%)		135 (55%)	103 (50%)	
	No, recent migrant (<2 years)	9 (11%)	28 (14%)		30 (12%)	29 (14%)	
Ethnicity	Bangladeshi	1 (1.2%)	6 (2.9%)	0.02	4 (1.6%)	5 (2.5%)	0.42
	Black-African	15 (19%)	62 (30%)		65 (26%)	63 (31%)	
	Black-Caribbean	2 (2.5%)	9 (4.4%)		10 (4.1%)	6 (2.9%)	
	Black-Other	5 (6.2%)	3 (1.5%)		11 (4.5%)	5 (2.5%)	
	Chinese	0 (0%)	3 (1.5%)		2 (0.81%)	4 (2.0%)	
	Indian	21 (26%)	50 (25%)		53 (22%)	54 (26%)	
	Pakistani	1 (1.2%)	13 (6.4%)		15 (6.1%)	12 (5.9%)	
	White	17 (21%)	25 (12%)		34 (14%)	26 (13%)	
	Other	19 (23%)	33 (16%)		52 (21%)	29 (14%)	
Sex	Male	59 (73%)	126 (61%)	0.07	164 (66%)	106 (52%)	<0.01
	Female	22 (27%)	79 (39%)		83 (34%)	98 (48%)	
Site of disease	Pulmonary	77 (95%)	179 (87%)	0.05	211 (85%)	150 (74%)	<0.01
	Non-pulmonary	4 (4.9%)	26 (13%)		36 (15%)	54 (26%)	
Social Risk Factor	History of homelessness	7 (8.6%)	14 (6.9%)	0.61	15 (6.2%)	9 (4.5%)	0.45
	History of imprisonment	5 (6.3%)	6 (3.0%)	0.20	10 (4.1%)	4 (2.0%)	0.20
	History of drug use	11 (14%)	19 (9.4%)	0.30	21 (8.6%)	12 (6.0%)	0.29
	Alcohol misuse	7 (8.9%)	11 (5.5%)	0.30	15 (6.3%)	7 (3.5%)	0.19
Age	15 years old or over	79 (98%)	192 (94%)	0.19	216 (87%)	148 (73%)	<0.01
	Under 15 years old	2 (2.5%)	13 (6.3%)		31 (13%)	56 (27%)	

The analyses involving strain typing data (objective 1) only included index cases that had a strain typed isolate and at least one contact who also had a strain typed isolate. Percentages are column percentages except for the total row. P-value is chi-squared p-values for differences between groups.

### Pairs of index cases and contacts with indistinguishable isolates

Overall, 67/85 (79%) of contacts who were diagnosed with TB had indistinguishable isolates from their index case. This was similar across a range of clinical and demographic factors relating to both the index case and the contact (Table 2). For pairs in which both case and contact had a social risk factor, 11/11 (100%) pairs had indistinguishable isolates (compared to 55/70 (75%) where either or both had no social risk factors, *P* = 0.07). For all other factors the p-value for the association with indistinguishable isolates was above 0.1.

**Table 2:** The proportion of contacts diagnosed with TB who share a strain with their index case.

Risk factors	(Number pairs with indistinguishable isolates)/(total number of pairs) (%)		p-value
Overall	67/85	(79%)	N/a
Index case pulmonary	65/81	(80%)	0.15
Index case extrapulmonary	2/4	(50%)	
Index case smear positive pulmonary	54/68	(79%)	0.75
Index case smear negative pulmonary	10/12	(83%)	
Index case UK born	26/30	(87%)	0.19
Index case non-UK born	41/55	(75%)	
Contact UK born	30/35	(86%)	0.19
Contact non-UK born	37/50	(74%)	
Both index and contact UK born	20/23	(87%)	0.26
Either index or contact or both non-UK born	47/62	(76%)	
Both index and contact non-UK born	31/43	(72%)	0.12
Either index, contact or both UK born	36/42	(86%)	
Both index and contact have one or more social risk factors	11/11	(100%)	0.06
Either index, contact or both have no social risk factors	55/70	(75%)	
Child contact	10/11	(91%)	0.29
Adult contact	57/74	(77%)	
Index case female	17/23	(74%)	0.50
Index case male	50/62	(81%)	
Contact female	30/38	(79%)	0.98
Contact male	37/47	(79%)	
Both index and contact female	7/10	(70%)	0.76
Both index and contact male	27/34	(79%)	
Index and contact different sex	33/41	(80%)	

Only index cases and contacts who both had typed isolates are included. The denominator is the number of case-contact pairs for whom that risk factor applies and the numerator is the number of these that have indistinguishable isolates.

If we remove pairs for whom the contact was listed as presenting through a route other than contact tracing, the proportion who have indistinguishable isolates was similar at 48/61 (79%). If we include pairs for whom the contact accessed care prior to the index, the proportion is slightly lower at 85/112 (76%), and the association between a higher proportion of pairs with indistinguishable isolates and the presence of social risk factors is weaker. Removing pairs which contain contacts already named in another pair did not change the results.

### Timescales of contact tracing

The median time interval between episode of care start dates for index and contact was 42 days (interquartile range (IQR): 16, 96). The time interval was slightly shorter for pulmonary index cases (41 days, IQR: 16, 96) compared to non-pulmonary index cases (56 days, IQR: 15, 103), and shorter for smear positive pulmonary index cases (37 days, IQR 14, 91) compared to smear negative pulmonary index cases (47 days, IQR 20, 96); the significance level of these differences was *P* = 0.12 and *P* = 0.57 respectively. There was also no evidence of a difference when comparing smear positive pulmonary, smear negative pulmonary and non-pulmonary index cases (*P* = 0.25). The median time interval was 42 days (IQR: 14, 96.5) among contacts diagnosed with pulmonary TB, and 47 days (IQR: 19.3, 94.5) for non-pulmonary contacts (*P* = 0.69).

**Figure 2:**
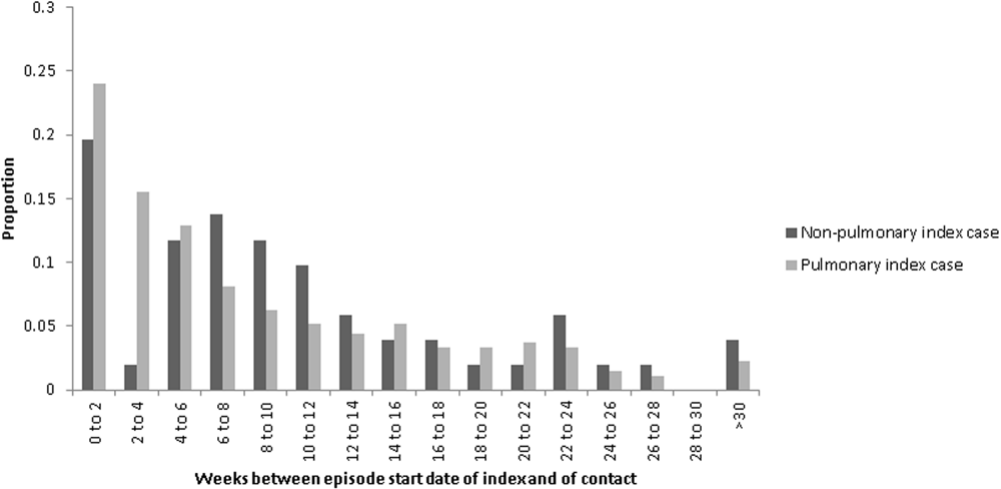
Distribution of the time in weeks between the episode of care start date of an index case and that of the contact, delineated by the site of disease of the index. Ranges include the upper bound, and exclude the lower bound, after the first bar.

This time interval between accessing care had a positively skewed distribution (Fig. 2) with most contacts accessing care within six weeks (52% for pulmonary index cases, and 33% for non-pulmonary index cases) and 39% (122/314) of all contacts first accessing care within one month of their index.

Contacts that were UK-born or recent migrants (entered within two years; numbers were small in this group) (aOR: 2.0 [1.2, 3.4] and 3.9 [1.3, 12] respectively) were more likely to be identified and access care within six weeks of their index case (Table 3), compared to longer-term migrants. Adult contacts (aged greater than 14 years) (aOR: 0.38 [0.23, 0.65]) were less likely to have a short investigation compared to children.

**Table 3:** Comparison of characteristics of contacts whose episode of care start date is six weeks or less after their index case with those of contacts whose episode of care start date is more than six weeks after that of their index case.

Factor		Number (%) of contacts with short or long time intervals between index case and contact accessing care		p-value	Adjusted odds ratio for investigation being short (95%confidence interval)
		six weeks or less	More than six weeks		
Total		142 (51%)	138 (49%)	N/a	N/a
UK-born?	Yes	78 (55%)	55 (40%)	<0.01	2.03 [1.24, 3.36]
	No	51 (36%)	77 (56%)		1
	No, recent migrant (<2 years)	13 (9.2%)	5 (3.7%)		3.88 [1.29, 11.7]
Ethnicity	Indian	25 (18%)	33 (24%)	0.06	1
	Black-African	52 (37%)	33 (24%)		1.95 [0.98, 3.89]
	White	24 (17%)	17 (12%)		1.59 [0.69, 3.62]
	Other	41 (29%)	55 (40%)		0.90 [0.46, 1.77]
Sex	Male	78 (55%)	73 (53%)	0.57	1.15 [0.71, 1.85]
	Female	64 (45%)	65 (47%)		1
Site of disease	Pulmonary	98 (69%)	87 (63%)	0.98	1.28 [0.78, 2.12]
	Non-pulmonary	44 (31%)	51 (37%)		1
Social Risk Factor	History of homelessness	3 (2.1%)	4 (3.0%)	0.66	0.71 [0.15, 3.28]
	History of imprisonment	2 (1.4%)	6 (4.4%)	0.20	0.34 [0.07, 1.74]
	History of drug use	7 (5.0%)	5 (3.7%)	0.60	1.44 [0.44, 4.75]
	Alcohol mis use	3 (2.1%)	4 (3.0%)	0.56	0.63 [0.14, 2.89]
	Any social risk factor	11 (7.9%)	11 (8.4%)	0.83	0.91 [0.38, 2.19]
Age	15 years old or over	75 (53%)	99 (72%)	<0.01	0.46 [0.28, 0.76]
	Under 15 years old	67 (47%)	39 (28%)		1

Percentages are within-group column percentages except for the total row. All odds ratios are adjusted for the site of disease of the index except for site of the disease of the contact, where it is omitted due to collinearity.

## Discussion

Our analysis estimates almost 80% of contacts diagnosed with TB and strain-typed in London are part of a recent transmission event involving the index case and the contact. This implies that 20% of contact investigations that find new cases do so even though no transmission has occurred between the index case and their contact. When both the case and the contact had one or more social risk factors, recent transmission was more likely to have occurred. The median time-interval between index and contact starting care was six weeks (42 days). Contacts who were adults (compared to children) or non-UK born migrants who entered >2 years previously (compared to UK born) were more likely to have an interval longer than six weeks. Contacts with social risk factors were not associated with delayed intervals of longer than six weeks.

A limitation of this study was the small number of pairs (85) where cases and contacts had strain typing results (21% of 406 pairs with strain-typed index cases; 12% of all 697 pairs). As a result, our analysis may have lacked power to discern all associations. In addition, findings may not be generalizable to other TB patients as for the included pairs the index cases were almost all pulmonary (95%), were more often of white or other ethnicity, more often UK born (35%), male (73%) and were almost all (98%) adults. We may have over-estimated the contribution of social risk factors as a result of this inclusion bias.

We may have overestimated the proportion of indistinguishable isolates compared to a higher resolution method such as whole genome sequencing (WGS)^13^. However, the combination of microbiological and epidemiological links is good evidence that patients with indistinguishable isolates represent recent transmission.

We were also not able to include 176 pairs where cases were identified through contact tracing, but the contact was not linked in the LTBR.

The proportion of index cases and contacts in London with indistinguishable isolates (79%) was higher than estimates of 70–71% in three previous studies in the United States of America^6–8^. The first of these studies found that pairs with unconfirmed transmission were more likely to include smear-negative source cases and a foreign-born secondary case, and less likely to include secondary cases under the age of 15 years, compared to pairs with confirmed transmission; the significance level of these relationships in our study was 0.75, 0.19 and 0.29 respectively. A recent UK-wide study found that 75% of pairs of cases with the same address had indistinguishable isolates^14^, supporting results presented here. Other recent studies based on MIRU VNTR typing data estimated the proportion of all cases due to recent transmission to be 34% in London and 10% in North-West England^15,16^. While these results are not directly comparable with ours, both findings support the notion that contacts of new cases of TB are more likely to have been recently exposed to tuberculosis than other TB cases, and so contact tracing is an essential tool to identify individuals at increased risk of disease.

A recent study in France found the mean period from index notification to completion of contact screening was 48 days^9^. A previous modelling study used a value of a quarter of a year (in rural Saskatchewan, Canada: a setting with greater barriers to screening than London)^17^, more than twice the median of 42 days found in London. A study in the Netherlands estimated the incubation period of TB cases (with any site of disease) to be around 1.3 years (95% CI: 1.1–1.4), with 30% of cases having an incubation period of less than six months^18^. This suggests that, for pairs with pulmonary index cases where there has been recent transmission from index to contact, for every week that a contact investigation is shortened, it may be possible to find and prevent disease in $\sim 1 \% (=\frac{30 \% }{26\,\mathrm{weels}})$ of infected contacts. However, a modelling study found that shorter contact investigations had little population-level impact^17^.

That case-contact pairs with social risk factors were more likely to have indistinguishable isolates than those without, coupled with the higher prevalence of disease seen in those with social risk factors^19^, suggests ongoing transmission occurring in this group in London, supporting previous studies^15,20^. This supports the emphasis given to this group in national guidelines, in particular recommendations for a programme of active case-finding amongst homeless and drug-users using a mobile X-ray unit, and to coordinate contact investigations around those with social risk factors in locations frequented by the index^11^. The higher proportion of indistinguishable isolates amongst these pairs may be because the index cases were more likely to be infectious^19,21^ and for longer, or because of the contact’s increased susceptibility.

Previous analysis of contact tracing outcomes in London found the prevalence of active disease amongst contacts of pulmonary patients to be 2.6%^4^. Assuming that, as found here, 80% of contacts of pulmonary index cases that develop active TB do so following recent transmission from their index case, this suggests that 0.52% of the contacts of pulmonary cases developed disease without transmission having occurred between the case-contact pair. This proportion can be seen as the risk of TB disease in contacts of TB patients that comes from sources other than the known index, perhaps due to shared risk factors and/or community contacts. A study in London in 2006 found the prevalence of TB amongst the homeless population to be 0.79%, amongst problem drug users to be 0.35%, and amongst prisoners to be 0.21%. Our study suggests that, even after removing the effect of transmission from the known index case, the risk of TB in a close contact of a TB case is high when compared with other risk groups in London^19^. However, contacts may self-present more quickly than the aforementioned risk groups.

In London, some clinics aim to screen contacts of smear positive patients within two weeks of contact identification, and contacts of non-smear positive cases within six weeks. While some contacts will be identified subsequent to diagnosis of the index case after the building of a relationship between case manager and patient, it is possible that these targets are not met for some contacts: 12 pairs had a time interval of more than six months between index case and contact accessing care.

WGS of TB isolates has recently been rolled out across the UK^22^. This will enable studies looking at transmission and clustered cases to link cases more accurately, which will in turn enable greater understanding of transmission networks and better target the allocation of resources.

We only found two pairs with an index case with non-pulmonary TB in which probable recent transmission occurred, but there were only four typed pairs with non-pulmonary index cases in total. Further research to understand the impact of 2016 changes to guidance, which no longer recommends screening contacts of non-pulmonary, non-laryngeal index cases^11^, would be useful. The results presented here could be utilized in modelling studies to assess the impact of contact tracing in different groups. Improved linkage of contacts would enable future research to have sufficient power to find risk factors with greater significance for the types of contacts which are most likely to have been part of a transmission event.

Whilst those pairs with social risk factors are more likely to involve recent transmission, these contacts may also be harder to identify and reach^4^. This highlights the importance of services such as Find & Treat in identifying these patients in London^23^. While we have quantified the typical times between index cases and their contacts accessing care, our study was not able to estimate the impact of shortening contact investigations, and when this is greatest. Further work to quantify this would be useful, perhaps incorporating mathematical modelling as well as data on the infectiousness of contacts. Finally, our results show that on the whole contact tracing in London happens in a timely manner thanks to the great effort of healthcare staff.
